# The Use of Tactile Sensors and PIV Analysis for Understanding the Bearing Mechanism of Pile Groups

**DOI:** 10.3390/s18020476

**Published:** 2018-02-06

**Authors:** Zhijia You, Yulong Chen

**Affiliations:** 1 College of Civil Engineering and Architecture, Shandong University of Science and Technology, Qingdao 266590, China; youzj@sdust.edu.cn; 2 Department of Hydraulic Engineering, Tsinghua University, Beijing 100084, China

**Keywords:** model test, group pile, interaction, tactile sensor, PIV

## Abstract

Model tests were carried out in dry silica sand under pile loading and visualizing observation to investigate the behavior of a pile group. The pile group consisted of nine cylindrical model piles of 40 mm in diameter in most tests or three rectangular parallelepiped model piles in the visualizing observation. Pile spacings of 200 mm and 100 mm between pile centers were used in the models. Tactile sensors were installed to measure the pressure distribution in the ground and colored sand layer with particle image velocimetry (PIV) analysis to reveal the ground deformation in addition to strain gauges inside the model piles to investigate the interaction among group piles. The tests results showed that a narrower spacing between piles resulted in a wider affected area of the ground and the interaction was more significant below the tips.

## 1. Introduction

The behavior of piles is a nonlinear, three-dimensional soil-structure interaction problem and the resulting response is a function of the pile arrangement, external loads and mechanical properties of the pile and soil [1,2,3,4]. Leung et al. [5] found that the role of nonlinearity in pile interaction becomes more significant when the pile spacing is less than 2.5 times the pile diameter. It is expected that the group pile effect would become more significant while the spacing becomes narrower.

Previous studies on group pile effects [6,7,8] were conducted in clayey ground. A negative effect, in which bearing capacity of a pile group was less than the summation of that of a single pile, on the bearing capacity of pile group was reported. On the other hand, a positive effect, in which the bearing capacity of pile group would be greater than the summation of a single-pile capacity, was also reported by Doohyun and Junhwan [9].

In spite of this knowledge, the mechanism of the group effect is not clear yet. To understand the mechanism, the interaction between piles and ground should be studied. It is thus necessary to observe precisely the sand behavior around the piles in detail during group pile loading.

The group pile loading tests and visualizing observation of a pile group were conducted with advanced sensors; tactile sensors and particle image velocimetry (PIV) tools. The latter tests were conducted to observe the ground deformation during the penetration of group piles by observing the deformation of colored sand layers and applying PIV analysis to the pictures of the ground deformation.

## 2. Test Apparatus and Test Procedure

**Figure 1:**
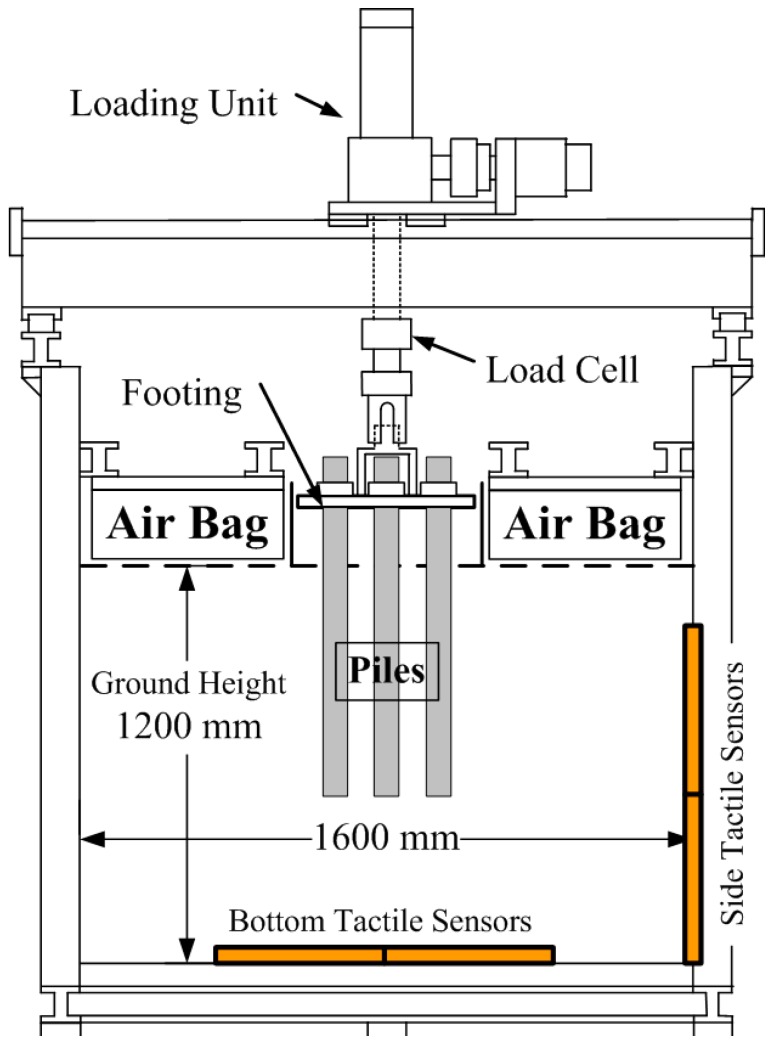
Cross section of test equipment.

Group pile loading tests and visualization tests were conducted in a large rigid soil tank as shown in Figure 1. Its internal dimensions were 1600 mm × 1600 mm (width) × 1200 mm (depth). The frontal wall of the tank was made of a transparent acrylic plate to observe ground deformation for PIV analysis. On the wall, a Teflon sheet was placed to reduce friction between soil and sidewall. On the top of this tank, a loading unit was installed, on which a load cell was set on the lower end of that unit, to measure the total axial loading on piles. The detail of this loading unit was described by Goto et al. [10]. Air bags were placed on the surface of the model ground with the exception of the testing area, to apply the confining pressure to the ground.

**Figure 2:**
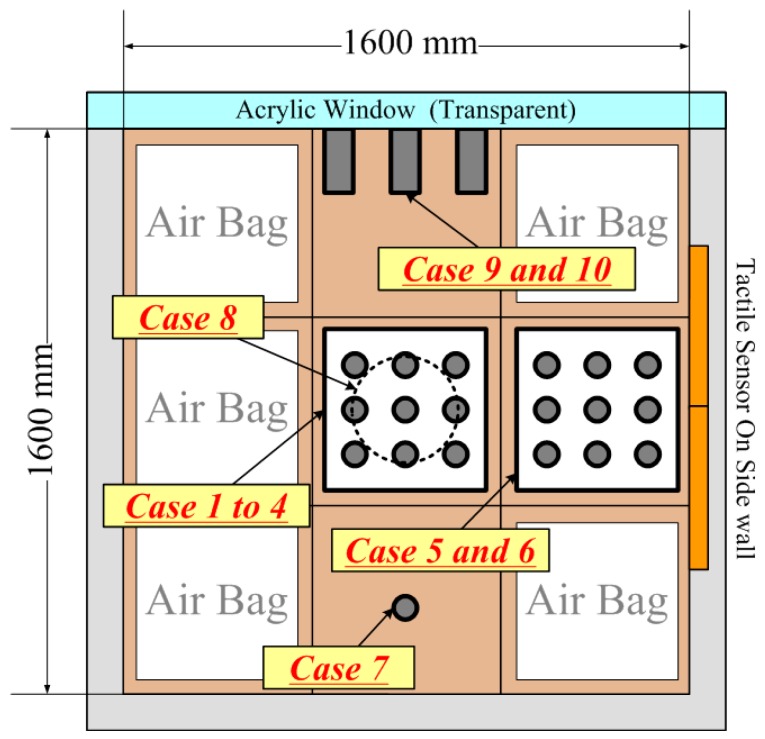
Test locations (top view of the soil tank).

All conducted test conditions are shown in Table 1. The testing locations in the table were shown in Figure 2. The test equipment was reset after every case. Model piles which constituted the pile group in the loading tests; from Case 1 to Case 6, were cylindrical in shape and made of aluminum, 40 mm in outer diameter, 4 mm in thickness, and 1000 mm or 1300 mm in length. The bottom of the piles was closed by a flat plate and the strain gauges were attached inside the pile at five levels along the piles and each level had four strain gauges to measure both the axial force and bending moments in two directions.

**Figure 3:**
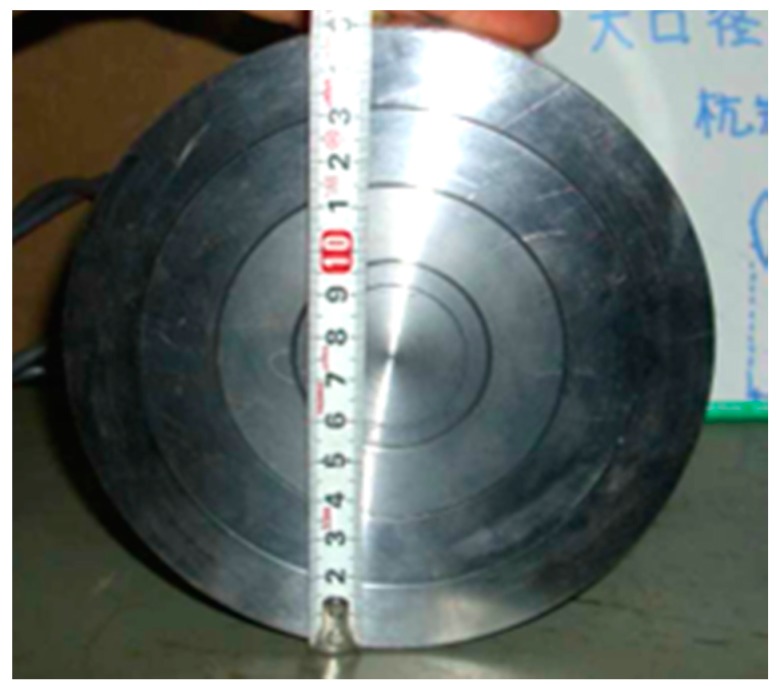
Annular load cells on the bottom of large pile.

For comparison with the group pile behavior, single pile loading tests were conducted with the same diameter of pile (Case 7); 40 mm in outer diameter, and a large diameter pile (Case 8); 150 mm in outer diameter, 10 mm in thickness and 1000 mm in length. The strain gauges were attached inside the both piles at the same location as in the constituting piles of the group pile. Moreover, the bottom of the large pile was closed by the load cell that was divided into annular 4 rings and the contact pressures were measured individually for each ring as shown in Figure 3.

In the visualization tests (Case 9 and Case 10), each model pile was a rectangular parallelepiped in shape, and made of aluminum, 40 mm × 80 mm in cross section, 4 mm in thickness and 1000 mm in length. The bottom of the piles was closed by flat plates and the strain gauges were attached inside the pile at top and bottom. Each level had four strain gauges similar to the cylindrical model piles.

Model ground was 1200 mm in height and made of air-dried Silica Sand No. 5; D_50_ = 0.523 mm, e_max_ = 1.09 and e_min_ = 0.66. It was constructed by the air pluviation method and manual compaction at every 150 mm deposition. The total amount of sand and the height of the ground were measured when sand was poured. The relative density calculated from the weight and height was around 90%. Such high relative density and the surcharge on the surface of the model ground were adopted to simulate the condition near the tip of the end bearing pile.

After the ground was built up below the initial height of the pile tips, the pile models were set on the ground. Each head of pile was fixed to a steel plate that is called “footing” in Figure 1 in group pile tests. After setting the piles, the ground was built again up to 1200 mm in height. Then the above-mentioned air bags were installed on the surface of the ground.

**Figure 4:**
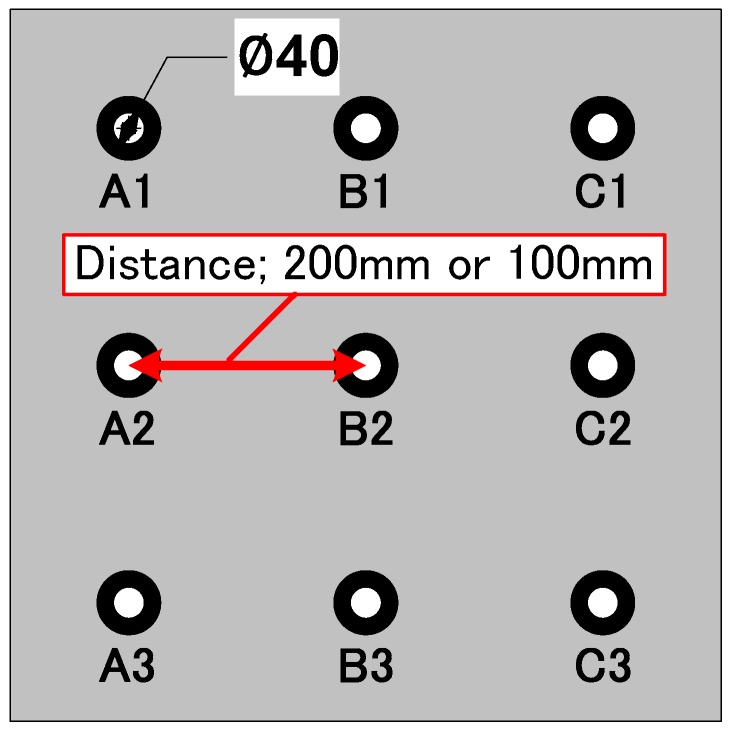
Pile layout of the group pile loading tests.

The group pile tests, Case 1 to Case 6, were performed with nine cylindrical piles (3 × 3) at the center or near the side wall of the soil tank. Two kinds of center-to-center spacing between piles were used: 5 times (200 mm) and 2.5 times (100 mm) diameter of piles. The plan view of the configuration of the pile group is shown in Figure 4. In each test, two kinds of pile loading were employed in order to evaluate the effect of induced soil fabric. First was individual loading in which each pile was separated from the footing and pushed down individually while other piles did not move. The sequence of individual pile loading is as follows: A 1,2,3 → B 1,2,3 → C 1,2,3. Namely, loading was given from A group to C group, and from #1 to #3 for every group. Another was group pile loading in which the footing was pushed down with all piles connected to the footing, which meant all piles were pushed down together. These tests were performed in displacement-control manner; the loading rate was 2 mm/min in individual loading and was 1 mm/min in group pile loading. Both individual and group pile loadings were continued until the settlement became 30 mm under a confining pressure. For measurements of the tactile sensors, these loading processes were interrupted for several minutes at every 10 mm settlement. After that, the confining pressure was increased to the next step and the individual and group pile loading were conducted again under the condition. The confining pressure was increased from 50 kPa to 200 kPa, with 50 kPa increments as shown in Table 1. Increasing pressures were applied on the surface of the ground to simulate a deeper pile embedment.

The single pile loading tests in Case 7 and Case 8 were conducted under the same conditions of the confining pressure; 50 to 200 kPa, and the loading rate; 2 mm/min, with the group pile loading tests. For measurements of the tactile sensors, these loading processes were also interrupted for several minutes at every 10 mm settlement of piles.

**Figure 5:**
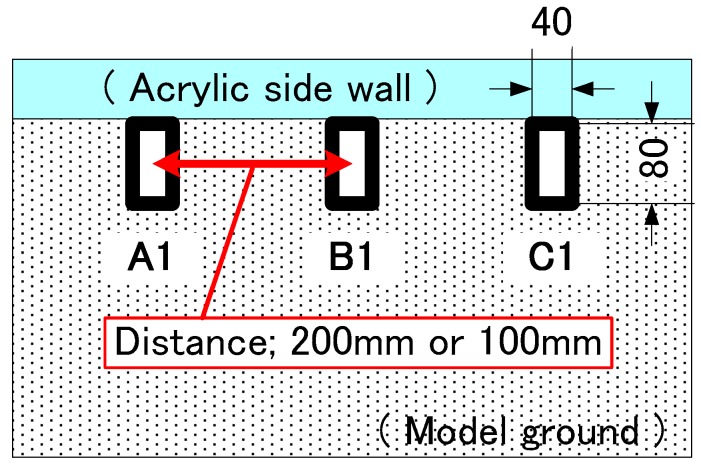
Top view of the group pile visualization tests.

The group pile visualization test, Case 9 and Case 10, were performed with three rectangular parallelepiped piles (3 × 1) behind the visualizing side wall (Figure 5). These tests reproduced a 2-dimentional model of a group pile and two kinds of center to center spacing were used such as the group pile; 200 mm and 100 mm. The plan view of the configuration of the pile group is shown in Figure 5. In both visualization test conditions, only group pile loading tests were carried out under each confining pressure, by increasing and decreasing that pressure (50, 100, 150, 200, 200, 150, 100 and 50 kPa).

## 3. Behavior of Piles under the Group Pile Loading Test

### 3.1. Yielding Point of the Bearing Load

**Figure 6:**
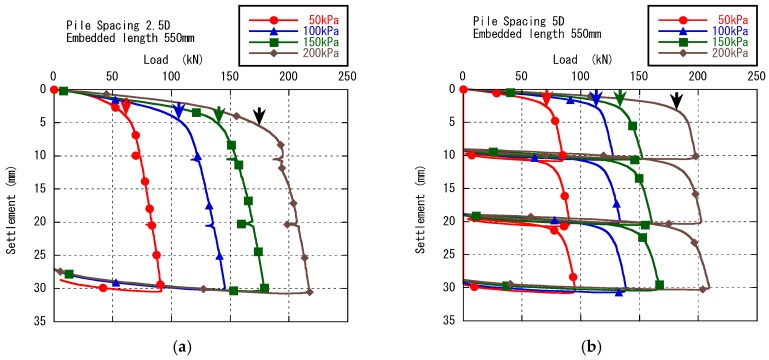
Load-settlement curves in (**a**) Case 1 and (**b**) Case 2.

**Figure 7:**
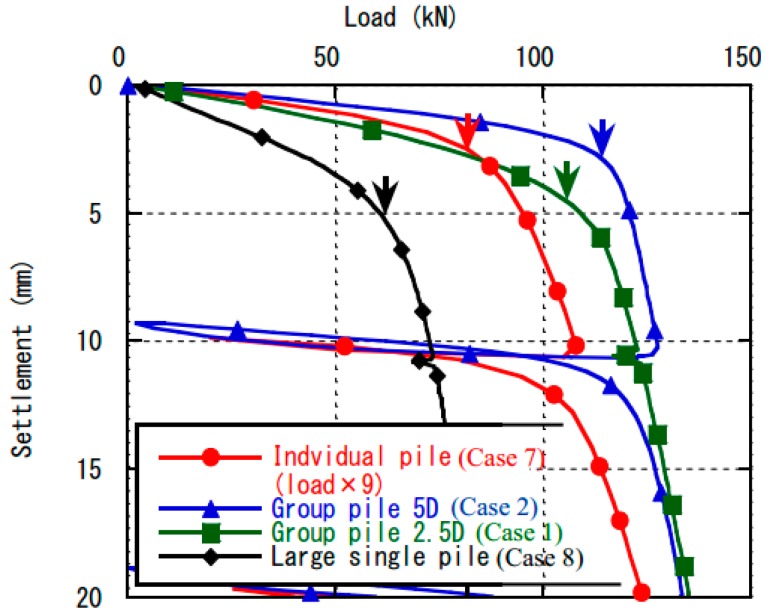
Yielding point of group pile, individual pile and large single pile at 100 kPa confining pressure.

The arrows in Figure 6 and Figure 7 indicate the yielding points, which were derived from the two tangent lines drawn at the beginning and final linear part of the load-settlement curves. The intersection of the bisector of the two tangent lines and the curve was defined as the yielding point. Although the yielding point depicted herein is a bit scale sensitive, the method provides an overall impression of ground status. The semi-log scale provides a better observation of the load-settlement relationship when the pile settlement is small.

Figure 6 shows the relationships between total bearing load measured by load cell and settlement during the group pile loading phase in Case 1 and Case 2. The load-settlement curves were classified and presented by confining pressures in all cases. All the yielding points of the curves were marked by arrows in the figure. The settlement at yielding points with 2.5D pile spacing are greater than that of 5.0D pile spacing under each confining pressure.

Figure 7 shows the load-settlement curves during the group pile loading tests at the confining pressure of 100 kPa in Case 1 and Case 2. The results of single pile loading test with the same diameter pile in Case 7 and the single pile loading test with large diameter pile in Case 8 under the same confining pressure were also plotted and compared in this figure. Load of the single pile with the same diameter in this figure was multiplied by nine, which is the number of piles, for comparison. The yielding points of the single pile tests were also marked by arrows in the figure.

Settlements at the yielding point of the single pile loading tests with the large diameter pile (Case 8) were much greater than that of the single pile loading test with the smaller diameter pile (Case 7).

Settlement at the yield point of the group pile loading test of 5D pile spacing (Case 2) was almost identical with that of the single pile loading test with the smaller diameter pile (Case 7). On the other hand, the settlement of group pile with 2.5D pile spacing (Case 1) was similar to the settlement of the single pile loading test with large diameter pile (Case 8). These settlement tendencies at yielding point seem to show that the pile group of 2.5D pile spacing behaved as one block or as a large pile, while the piles in group of 5D spacing behaved more independently.

### 3.2. Load Distribution between Each Pile

**Figure 8:**
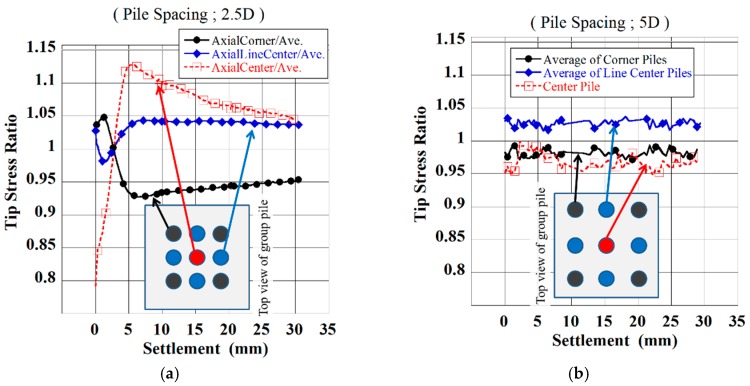
Tip resistance ratio among individual piles with spacing (**a**) 2.5D and (**b**) 5D.

**Figure 9:**
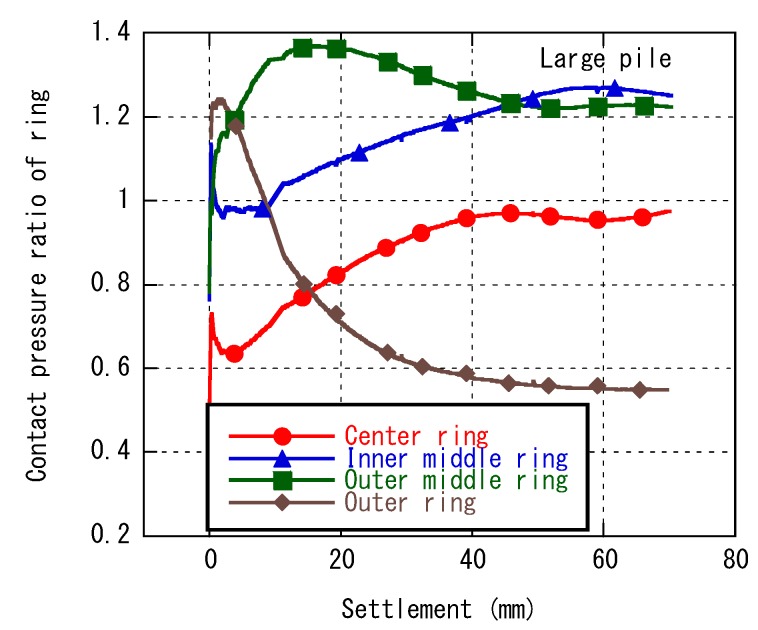
Tip resistance ratio on the bottom of large pile.

Figure 8 shows the mean tip resistance changing with the location of pile–central, central of outer line, and corner, normalized by the total mean tip resistance under the confining pressure of 50 kPa (Case 1 and Case 2). With the wider spacing, the ratio of each pile was almost equal to unity throughout the loading. It suggests that each pile behaved independently and that the interaction was insignificant. In contrast, for narrower spacing, the ratio changed with the penetration. The load concentration shifts from corner piles to center piles. Figure 9 shows the tip resistance changing with the location on the bottom of the large pile (Case 8), normalized by the total mean tip resistance. This tip resistance distribution was measured by annular load cells which were installed at the bottom of large pile.

The stress concentration also shifts from the edge to the center of the pile. This finding suggests that the significant interaction occurs for narrower spacing and these piles behave as one large block. For details, refer to Goto et al. [10].

## 4. Application of Advanced Sensors

To further understand the mechanism of pile-to-pile interaction, it is necessary to observe the ground behavior during the group pile loading. Thus, the advanced sensors were installed in the model ground.

### 4.1. Tactile Sensors

#### 4.1.1. Tactile Sensors Description

**Figure 10:**
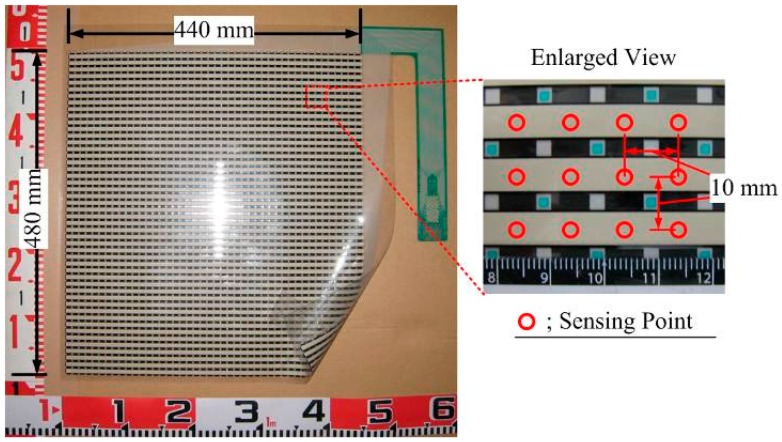
Tactile sensor sheet.

The tactile sensor (Figure 10) manufactured by Nitta Corp. (Osaka, Japan) were installed both at the bottom and the side wall of the soil tank. The advantage of this sensor is the ability to measure the distribution of normal pressure. Although earth pressure transducer is often used, the device cannot demonstrate the stress distribution and hence, is not relevant for the present research purpose, to discuss the effect of group pile on the stress transfer.

**Figure 11:**
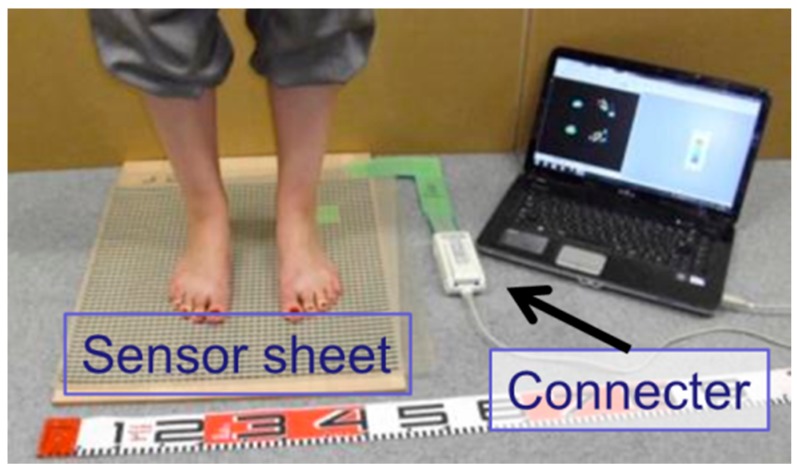
The sensing unit of tactile sensor sheet.

**Figure 12:**
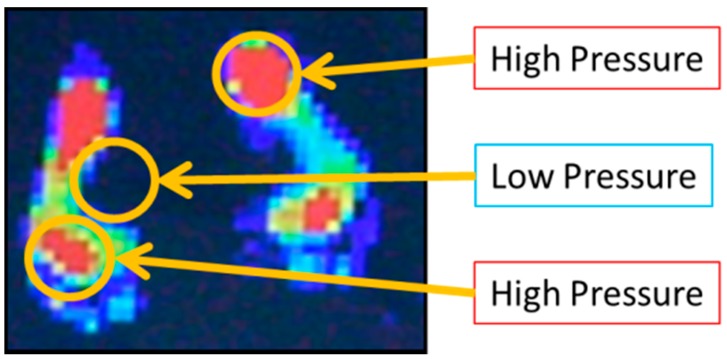
The pressure distribution measured by the tactile sensor.

Table 2 presents the specifications of tactile sensor. The sensor consists of two thin PET sheets and the inner side of each PET sheet contains rows and columns of resistive ink. At the point of grid, where rows and columns of the ink overlap, the applied forces are measured as the change of the electric resistance between rows and columns of ink. The sensor sheet covered 440 mm × 480 mm area, containing 44 rows and 48 columns of ink resulting in 2016 sensing cells spaced at 10 mm at centers in each direction. The system of tactile sensors includes this sheet sensor, a connecter and software as Figure 11 shows. The connecter reads the resistance value consecutively from the sensor sheet and the pressure distribution is calculated by the program as Figure 12 shows. In this example of the pressure distribution, the red part means higher pressure and the blue part the lower. The original data to show the pressure distribution has the definite pressure value at each sensing points. By changing the connecter, the data of pressure distribution are gained from several sheets and these data are synthesized by the supplied software.

The disadvantage of this sensor is an error caused by friction force on the sensor. It was shown that the value of normal stress would be reduced 20–50% when the shear force occurred [11]. That is why a Teflon sheet was installed between the tactile sensors on the side wall and the model ground to remove the friction against that sensor. Another possible disadvantage may be that pore fluid pressure cannot be measured by this sensor.

#### 4.1.2. Vertical Pressure Distributions

**Figure 13:**
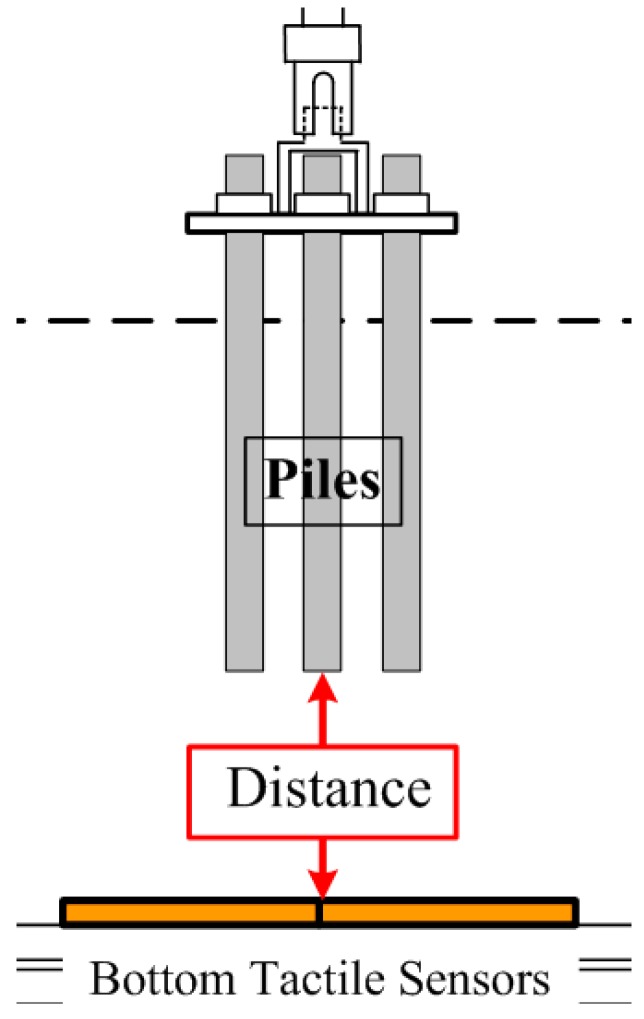
The distance from the pile tips to the tactile sensors.

**Figure 14:**
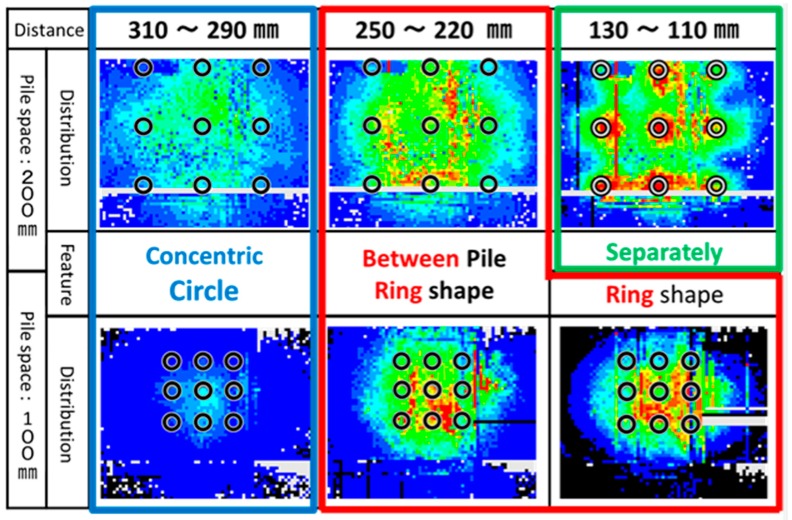
The vertical pressure distribution on the bottom of the soil tank.

As shown in Figure 13, “Distance” in the figure means the distance from the pile tips to the tactile sensor at the bottom when the pressure was measured. Figure 14 shows the representative distribution of normal pressure at the bottom of the soil tank measured by tactile sensors (Case 3 and Case 4). The pressure value in the figure is the difference between pre- and post-loading conditions.

The pressure distribution would be classified into three types according to the distance. First, when the distance between pile tips and the sensors at the bottom is large enough; more than 290 mm in both test cases, the higher pressure (red color) occurred below the central pile and the pressure decreased concentrically in the radial direction (becoming blue) for both narrower and wider spaces. Second, with distance becoming smaller, the maximum pressure shifted to the zones between piles and formed a circular distribution. The pressure below the central pile did not achieve the maximum. This kind of distribution is defined as toroidal distribution. This distribution also arose in both narrower and wider space group pile loading tests. Third, with distance becoming even smaller, the distribution changed only in case of wider spacing. The high pressure area is located just under the piles separately. On the other hand, the stress distribution under narrower spacing kept the toroidal distribution at this distance.

These changing of the vertical pressure distributions under group pile loading were detected for the first time by measuring with the tactile sensors. As the distance between piles decreased, the stress below center pile is much higher than the other piles. This distribution reveals the effects of the interaction in the ground under group pile loading. Therefore, these three kinds of distribution were compared with the two kinds of superposition of the pressure distribution to investigate the effect of the interaction.

**Figure 15:**
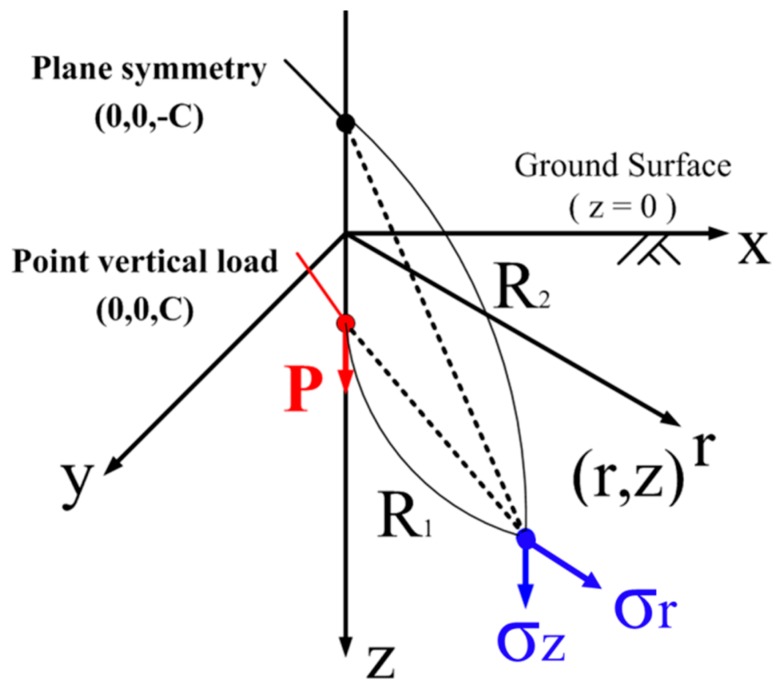
Computational condition of the Mindlin solution.

First superposition was made of the elastic solution calculated by the first solution of Mindlin. Mindlin’s elastic solution shows the normal and horizontal stress distribution when a vertical point load is applied on the elastic half space as Figure 15 shows. Equation (1) shows the solution of the normal stress distribution:

(1) $$\begin{array}{l} \sigma_{z}=\frac{P}{8\pi \left(1-\upsilon \right)}\{-\frac{\left(1-2\upsilon \right)\left(z-c\right)}{R_{1}^{3}}+\frac{\left(1-2\upsilon \right)\left(z-c\right)}{R_{2}^{3}}-\frac{3{\left(z-c\right)}^{3}}{R_{1}^{5}}\\ -\frac{30cz{\left(z+c\right)}^{3}}{R_{2}^{7}}-\frac{3\left(3-4\upsilon \right)z{\left(z+c\right)}^{2}-3c\left(z+c\right)\left(5z-c\right)}{R_{2}^{5}}\} \end{array}$$

where, *P* is the point vertical load in the ground; *ν* is the Poisson ratio (assumed as 0.3); *z* is the depth from the ground surface; *c* is the depth of the point vertical load; *R*_1_, *R*_2_ are Distance from the point load or the symmetry of the point load, respectively.

The pile tip load measured by strain gauges in tests was considered as a point load “*P*”. The vertical stress distribution was calculated for each point load at the location of the pile tips separately, and then assembled to calculate the stress distribution under group pile loading at the same depth as the tactile sensor.

**Figure 16:**
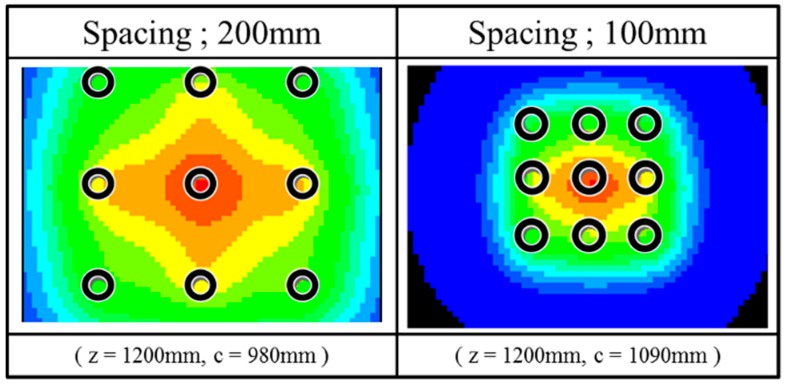
The superposition of the elastic solution.

A significant difference between the experimental data and the superposition of the elastic solutions occurred at the toroidal pressure distribution. Figure 16 shows the superposition of the elastic solutions of vertical stress in the ground at 220 mm (in case of 200 mm pile spacing) or 110 mm (in case of 100 mm pile spacing) distance from the pile tips where the toroidal pressure distribution appeared in the actual experiments. The locations under the piles are shown by the black circles in this figure. The superposition could not reproduce the feature of actual pressure distribution profile under group pile loading-toroidal profile, central column and right below in Figure 14.

**Figure 17:**
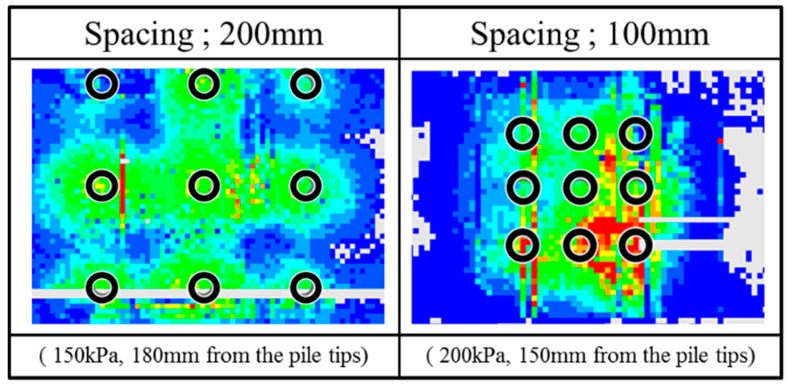
The superposition of the measured pressure distribution under individual loading.

The second one is the superposition of the pressure distribution measured under individual loading. The individual loading tests were conducted before the group pile loading tests at every confining pressure and the bottom pressure distribution during loading each pile was measured separately. Figure 17 shows the superposition of the individual pressure distribution at the most similar distance with that when the toroidal profile was measured under group pile loading. The superposition could not reproduce the feature of measured pressure distribution profile as well.

The incompatibility of the real pressure distribution with the two kinds of superposition suggests that the interaction of each pile would be strong in this area where toroidal pressure distribution profile showed.

Because the wider spacing pile group also shows the toroidal pressure distribution as well, the wider group spacing generated the interaction of piles. This consideration is different from the observation based on the bearing load or tips stress distribution among piles.

One of the reasons for this difference may be the location of the area where this toroidal pressure distribution occurs. In case of wider spacing, the toroidal pressure distribution occurred at a distance from the pile tips. That is why the interaction in the ground would not affect the bearing capacity or tip resistance distribution of the wider spacing. In contrast, the toroidal distribution occurred near the pile tips in case of narrow spacing. This would affect the behavior of the piles; as a result, the yielding point was different from the superposition of the single pile loading (Figure 7) and the tip stress distribution showed the changing with distance as shown in Figure 8.

#### 4.1.3. Horizontal Pressure Distributions

**Figure 18:**
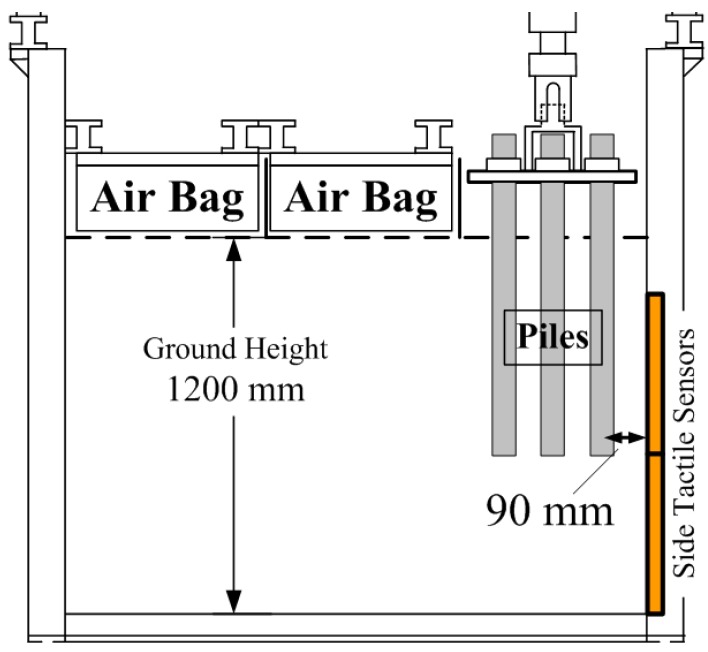
Cross section of test equipment in Case 5 and Case 6.

**Figure 19:**
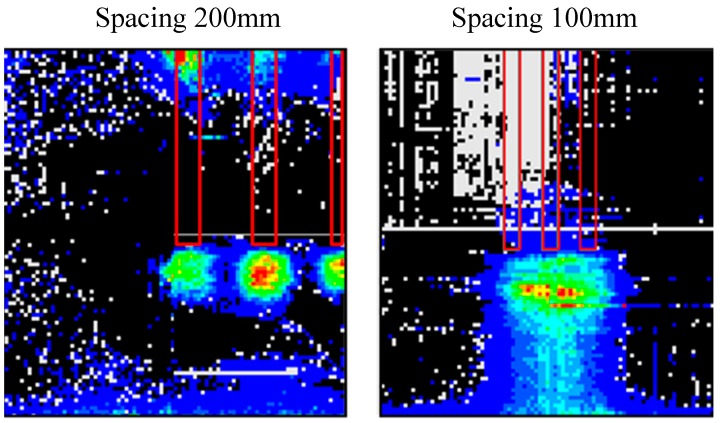
Tip horizontal pressure distribution under group pile loading.

The horizontal pressure distribution was measured by tactile sensors placed on the side wall when group pile loading tests were conducted near the side wall (Case 5 and Case 6) as Figure 18 shows. The side wall was 90 mm away from the center of the nearest pile in a group. Figure 19 shows the horizontal pressure distribution at the confining pressure of 100 kPa. The pressure value is also the difference between pre- and post-loading conditions. The locations of the piles are shown by red line in the figure.

**Figure 20:**
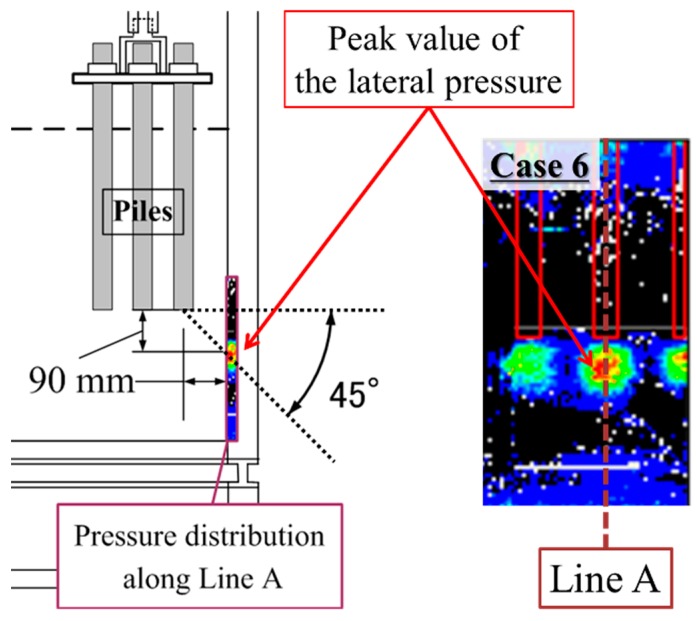
The position where the peak value of lateral pressure occurred.

In case of the wider spacing pile group, higher pressure occurred under each pile separately and the shape of each distribution was concentric. The highest pressure in each distribution occurred around 90 mm below the pile tips. This suggests that the highest pressure occurred around 45 degrees obliquely downward from the pile tip as Figure 20 shows. This profile is compatible with the superposition of the results measured during the individual loading tests or the elastic solutions calculated by Mindlin 2nd solution.

On the other hand, in case of narrower spacing pile group, a clumpy distribution was observed. This would support the point that the narrow spacing behave as one block because of the higher interaction among piles. Moreover, it was detected that the pressure changed area propagated downward in narrow spacing group pile as shown in Figure 19. This insists that the effective area of the ground below the piles would expand because of the interaction of piles. It would cause the larger deformation of the ground below the piles in narrow spacing group pile. That is why the settlement stiffness in narrower spacing pile group would decrease in elastic phase as shown in Figure 7.

### 4.2. PIV Analysis

**Figure 21:**
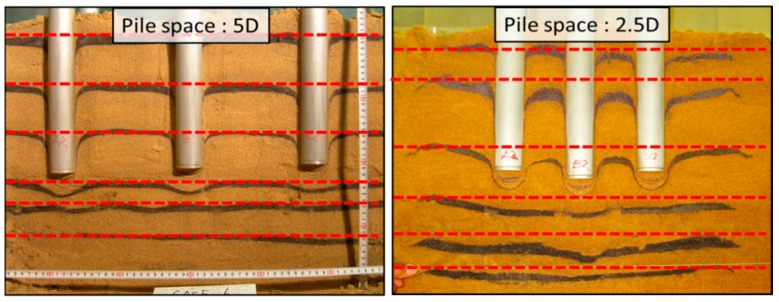
The ground deformation after the loading tests in Case 2 and Case 1.

The significant interaction in a group pile with narrow spacing was also studied from the viewpoint of ground deformation. Figure 21 shows the ground deformation after loading tests in Case 1 and Case 2.

**Figure 22:**
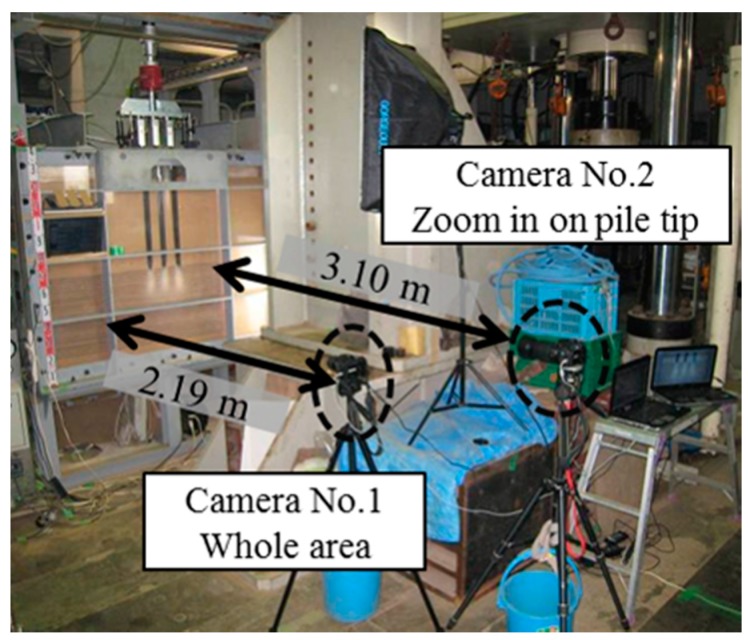
Observation system of the real-time ground deformation.

The dotted lines in the figures show the initial positions of each colored sand layer before loading tests. In case of the group pile with wider space, the layers deformed only below pile tips and the layers between piles remained at the original location. In contrast, in case of narrow space, the ground below the pile tips deformed in a contiguous convex way and the ground between the piles also deformed downward. These different shapes of the ground deformation according to the pile space suggest that the group pile with narrow space affected the ground as one block because of the interaction between piles. To further study this effect of the interaction on the ground deformation, real-time observation of the ground deformation through a transparent acrylic wall is conducted as shown in Figure 22. The group pile was simulated as the 2-D model in this visualization tests, by using three rectangular piles as shown in Figure 5. Colored sand layers were installed in the ground below the pile tips every 40 mm and the thickness of each layer was 20 mm. The progress of the deformation was recorded by two digital cameras (Nikon D60, Nikon Corporation, Tokyo, Japan). The records were taken at every 5 s automatically by the software (Nikon Camera Control Pro 2). One camera recorded the whole ground deformation. Another one zoomed in on the area below the pile tip; about 300 mm in width and 200 mm in height, by using a 200 mm telephoto lens. Two lights were installed to exclude shadows from the observation area.

The visual observation of the consecutive images in Case 9 and Case 10 detected that the deformation in one contiguous convex way started from the initial state of the penetration in case of narrow spacing group pile.

**Figure 23:**
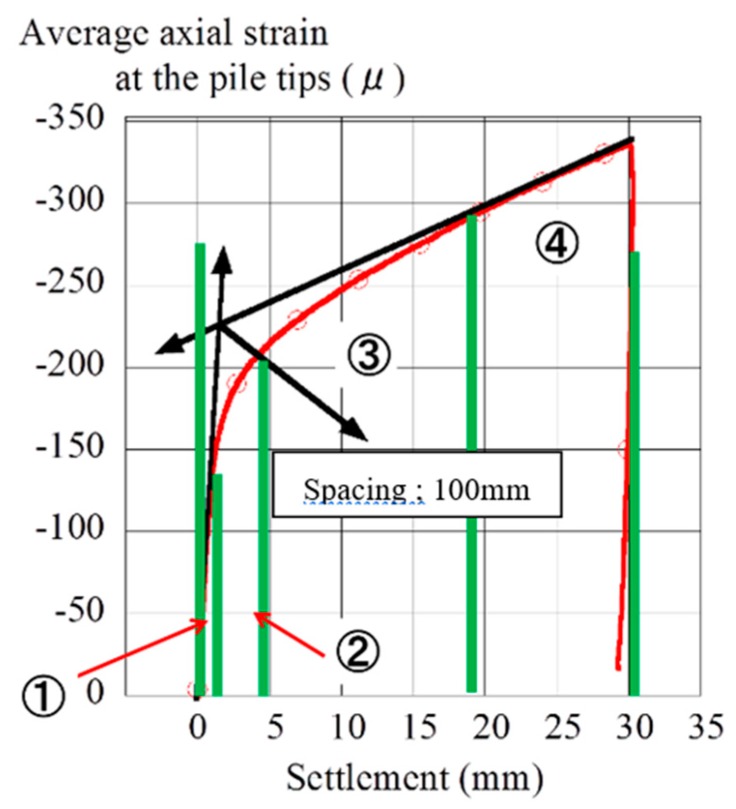
The four phases for PIV analysis.

To analyze this real-time deformation quantitatively, the PIV analysis was applied to these pictures by using GeoPIV [12]. Figure 23 shows the relationship between the averaged axial strain at the pile tips measured by the strain gauges inside the piles and settlement during the group pile loading under the confining pressure of 100 kPa. The result was split into 4 phases; 1-Elastic, 2-Before Yielding Point, 3-After Yielding Point, and 4-Plastic. The PIV analysis was applied for each phase individually. The analysis conditions for PIV are shown in Table 3.

**Figure 24:**
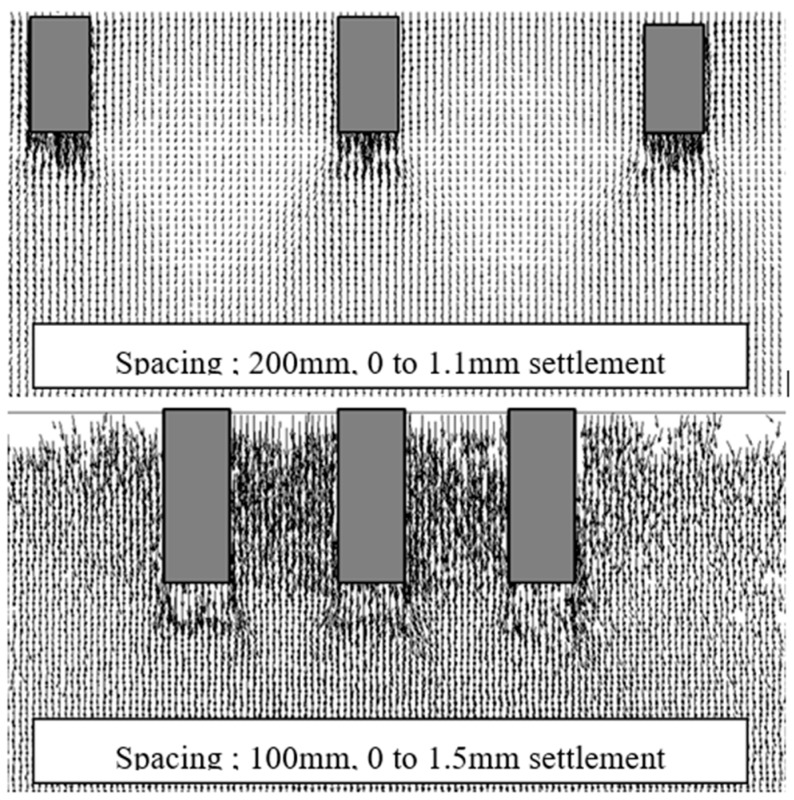
The PIV results in the elastic phase ①.

Figure 24 shows the results of PIV analysis in the elastic phase. The arrows in the figures show the vector of the ground displacement in the phase. In case of the group pile with wider space, the ground between piles did not deform except for the surface of the piles. In contrast, the ground between piles deformed downward with the piles in case of narrow space. These indicate that the group pile with narrow space affected the ground as one block because of the interaction.

**Figure 25:**
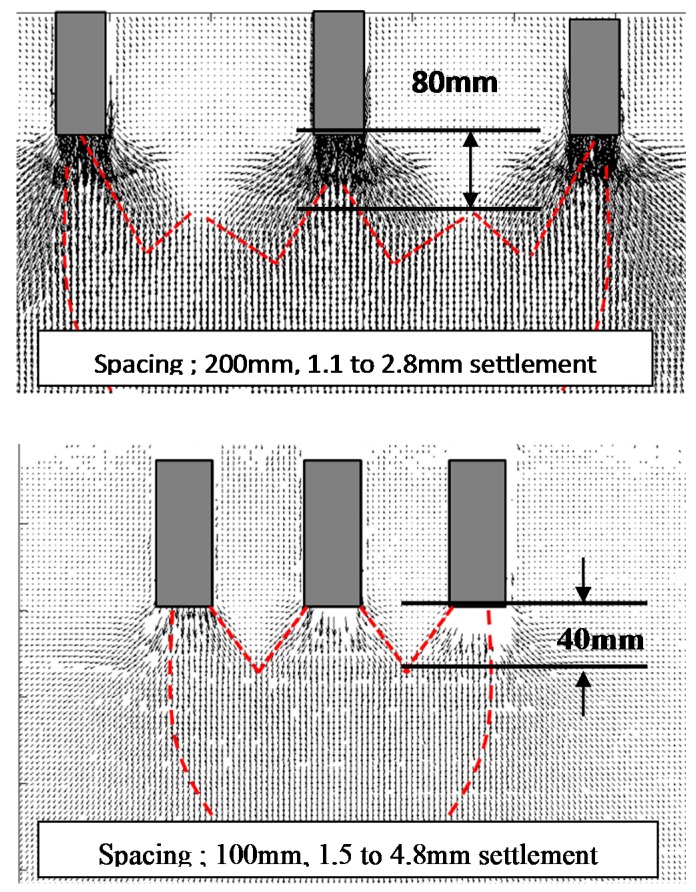
The PIV results in the phase before yielding point ②.

**Figure 26:**
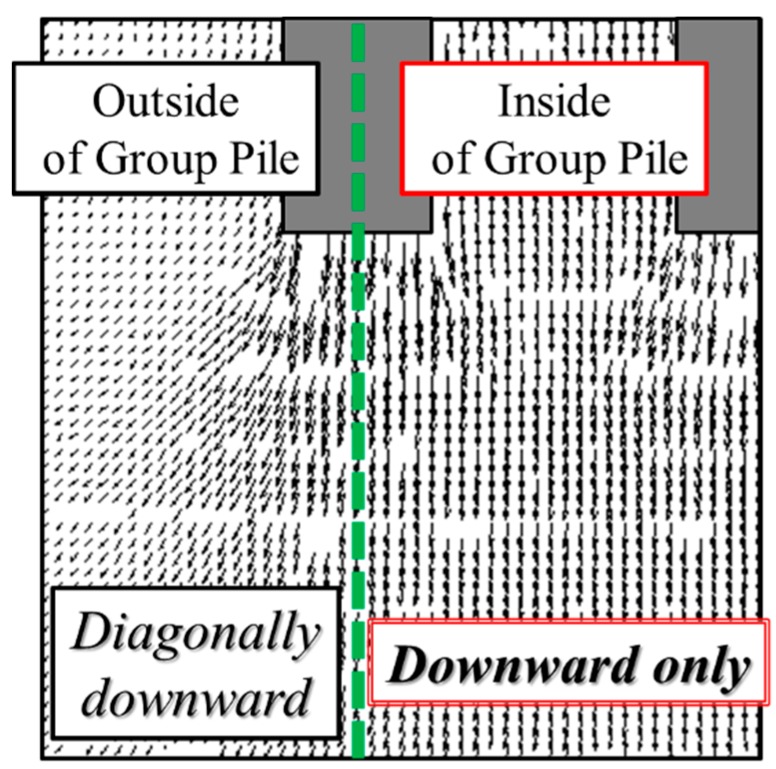
The different directions of the vectors inside and outside the group pile in phase ②.

Figure 25 shows the results of PIV analysis in the phase before yielding point and Figure 26 is the enlarged view of the result in case of narrow space. The dotted line in Figure 26 shows the center line of the outer pile and the right side of the line is the inside of the group pile. The directions of arrows are different between inside and outside of the group pile. The main direction of the arrows outside the group pile is diagonally downward. This profile would be similar with the profile which was observed during the single pile loading. In contrast, the main direction inside the group pile is downward. This profile would be caused by the interference of the ground deformation. Two retrorse diagonally downward vectors of ground deformation would collide and be translated to a downward vector. That is why the area of interaction is defined as the area where the downward vectors are dominant.

The interaction areas based on this definition are shown in Figure 25 by using the dotted line. The interaction areas were observed in both cases of the narrow and wide spacing group pile but the distance from the pile tips to the upper end of the area was different. In case of the narrow space, the upper end was 40 mm distance from the pile tips. On the other hand, in case of the wide space, the upper end was 80 mm distance from the pile tips. This difference would affect the behavior of the group pile. In the former case, the ground below the central pile was confined by this interference of the ground deformation. It would cause the higher ratio of the tip resistance at the central pile as shown in Figure 8. In contrast, the interaction area would not affect the behavior of the group pile because the distance from the pile tips to the upper end of interaction area was far in case of wider space. This observation is consistent with that of the pressure distribution on the bottom of the soil tank.

## 5. Conclusions

Vertical loading tests of a pile group and visualization tests were conducted. After comparing the bearing load and distribution of tip load among piles, the vertical and lateral pressure distribution and the ground deformation between two kinds of pile spacing: 5D and 2.5D, the following conclusions may be drawn:

(1) For a narrow space pile space, a group pile yields at a larger displacement and the apportionment of tip resistance among piles changed depending on the penetration length. These behaviors are consistent with that of large diameter pile. This suggests that the group pile with narrow space works as one block.
(2) Toroidal distribution was observed in the vertical pressure recorded by the tactile sensors. This form was not represented by simple superposition and implied the interaction of piles. This interaction would affect the behavior of a pile group as one block, if it occurred near the pile tip in case of the narrow spacing group pile.
(3) A clumpy distribution was observed below the piles by the tactile sensors on the side wall in narrower spacing pile group. This support the point that the group pile with narrow space works as one block. Furthermore, the downward propagation of lateral pressure distribution was also measured in wider area at the narrow space. This suggests the large deformation under the same penetration length and the settlement stiffness will decrease in narrow spacing.
(4) The consecutive ground deformation in a block way occurred in the elastic phase only in case of the group pile with narrow space. The interference of ground deformation affected the behavior of the group pile because the distance from the pile tips to the area was short enough.

## Figures and Tables

**Table 1:** All conducted test conditions.

Case No.	Aim of Tests	Test Location	Loading Rate	Pile Shape	Pile Length	Number of Piles	Spacing between Piles	Confining Pressure
1	Group pile loading test	Center	1 mm/min	Cylindrical diameter 40 mm	1000 mm	9 (3 × 3)	100 mm (2.5D)	50 kPa → 100 kPa → 150 kPa → 200 kPa
2							200 mm (5D)	
3	Vertical pressure distribution under group pile loading				1300 mm		100 mm (2.5D)	
4							200 mm (5D)	
5	Horizontal pressure distribution under group pile loading	Near the side wall			1000 mm		100 mm (2.5D)	
6							200 mm (5D)	
7	Single pile loading test	Near the back wall	2 mm/min			1		
8		Center		Cylindrical diameter 150 mm				
9	Group pile visualization tests	Near the front wall	1 mm/min	Rectangular parallel pipe width 40 × 80 mm		3	100 mm (2.5D)	50 kPa → 100 kPa → 150 kPa → 200 kPa → 150 kPa → 100 kPa → 50 kPa
10							200 mm (5D)	

**Table 2:** System specifications of tactile sensor.

Parameters	Values
Technology	Piezoresistive
Sensing Point Size	1″ × 1″ (25.4 mm × 25.4 mm)
Scan Speed	Up to 35 hertz
Temperature	10–40 °C
Accuracy	±10%
Repeatability	±2%
Hysteresis	±5%
Non-linearity	±1.5%

**Table 3:** The analysis conditions of PIV analysis.

Parameters	Values
Size of target mesh	24 pixel
Space between target mesh	8 pixel
Search range	250 pixel
Resolution	7360 × 4912 pixels
Time interval between pictures	20 s
